# Endometrial regenerative cells: A novel stem cell population

**DOI:** 10.1186/1479-5876-5-57

**Published:** 2007-11-15

**Authors:** Xiaolong Meng, Thomas E Ichim, Jie Zhong, Andrea Rogers, Zhenglian Yin, James Jackson, Hao Wang, Wei Ge, Vladimir Bogin, Kyle W Chan, Bernard Thébaud, Neil H Riordan

**Affiliations:** 1 Bio-Communications Research Institute, Wichita, USA; 2 Medistem Laboratories Inc, Tempe, USA; 3 Department of Surgery, University of Western Ontario, London, Canada; 4 Department of Pediatrics, University of Alberta, Edmonton, Canada

## Abstract

Angiogenesis is a critical component of the proliferative endometrial phase of the menstrual cycle. Thus, we hypothesized that a stem cell-like population exist and can be isolated from menstrual blood. Mononuclear cells collected from the menstrual blood contained a subpopulation of adherent cells which could be maintained in tissue culture for >68 doublings and retained expression of the markers CD9, CD29, CD41a, CD44, CD59, CD73, CD90 and CD105, without karyotypic abnormalities. Proliferative rate of the cells was significantly higher than control umbilical cord derived mesenchymal stem cells, with doubling occurring every 19.4 hours. These cells, which we termed "Endometrial Regenerative Cells" (ERC) were capable of differentiating into 9 lineages: cardiomyocytic, respiratory epithelial, neurocytic, myocytic, endothelial, pancreatic, hepatic, adipocytic, and osteogenic. Additionally, ERC produced MMP3, MMP10, GM-CSF, angiopoietin-2 and PDGF-BB at 10–100,000 fold higher levels than two control cord blood derived mesenchymal stem cell lines. Given the ease of extraction and pluripotency of this cell population, we propose ERC as a novel alternative to current stem cells sources.

## Introduction

Stem cells are undifferentiated cells that can replicate themselves without differentiating, and under specific conditions can differentiate into various specialized cell types. Stem cell therapy holds tremendous promise for repair and/or regeneration of aging and damaged tissue. Broadly speaking, stem cells can be divided into embryonic and adult types. While embryonic stem cells possess great ability to proliferate, the specific induction of their controlled differentiation has been elusive [1-3]. Additionally, embryonic stem cells possess the possibility of immune rejection of their differentiated progeny [4]. The fear of embryonic stem cells causing teratomas has also been a major obstacle to their clinical development [5]. Adult stem cells derived from tissues such as bone marrow [6], cord blood [7], adipose tissue [8] or the amniotic fluid [9] have demonstrated regenerative potential in a variety of diseases and degenerative disorders, however, these cells types are limited by: availability, invasiveness of extraction, and in some cases limited proliferative capacity. What is currently needed is a source of stem cells that overcomes these deficiencies, while not possessing the fear of karyotypic abnormalities during culture and possibility of oncogenesis.

The monthly preparation of the endometrium for receiving of the fertilized egg is associated with a period of hyperproliferation and angiogenesis [10]. The lining of the endometrium expands by 5–7 mm in thickness within each menstrual cycle [11]. Given this very rapid angiogenesis, a great amount of tissue remodeling, growth factor secretion, and endothelial sprouting must occur in a tightly regulated manner. It is known that in certain conditions of hyperangiogenesis, such as in cancer, stem cells with angioblast properties migrate from the bone marrow and actively participate in the angiogenic cascade [12]. Accordingly, we sought to investigate whether cells with stem cell like properties may be found in the menstrual blood during periods of menstruation.

We identified an adherent cell population possessing non-hematopoietic markers that effectively could be propagated for > 68 doublings while maintaining karyotypic normality and ability to differentiate into numerous tissues. These cells, which we have termed Endometrial Regenerative Cells (ERC), may be easily expandable and useful for females as a non-invasively obtained and ethically appropriate autologous stem cell alternative.

## Materials and methods

### Generation of endometrial regenerative cells

Menstrual blood was collected from a healthy female subject after menstrual blood flow initiated. Collection was performed in a urine cup and then transferred into a 5 ml tube with 0.2 ml amphotericin B (Sigma-Aldrich, St Louis, MO), 0.2 ml penicillin/streptomycin (Sigma 50 ug/nl) and 0.1 ml EDTA-Na2 (Sigma) in phosphate buffered saline (PBS). Mononuclear cells derived from menstrual blood were separated by Ficoll-Paque (Fisher Scientific, Portsmouth NH) according to the instruction and washed in PBS. Cells were subsequently cultured in a Petri dish (Corning, Acton, MA) containing DMEM medium supplemented with 1% penicillin/streptomycin, 1% amphotericin B, 1% glutamine and 20% FBS (completed DMEM). Media was changed the next day. Adherent cells were detached by trypsin and cultured in a T75 flask (Fisher Scientific, Portsmouth NH) at 1 × 10^5 ^cells. The cells were then subcultured and passaged twice a week. Cloning of cells was accomplished by plating cells at a concentration of approximately 1 cell per well in 96 well plates (Corning, Acton, MA).

### Phenotypic characterization

For fluorescent antibody cell surface staining cells were washed with HBSS+2%BSA two times and incubated with the specific antibody at concentrations recommended by the respective manufacturer. Cells were incubated for 20 min and analyzed either under fluorescent microscope or flow cytometry. The antibodies used were: CD markers, SSEA-4, Stro-1, HLA-ABC and HLA-DR. These were purchased from BD Pharmingen, Ancell, Stem Cell Technologies, eBioscience, Chemicom, Miltenyi Biotech and R&D Systems. For intracellular staining by the antibodies without any conjugate, cells were washed twice in Hank's solution with 2% BSA and fixed with 4% Formalin for 1 hour. Subsequently cells were washed twice in 0.5% Tween20 and 0.1% Triton X-100 in PBS (T-PBS). Primary antibodies were added to T-PBS at the concentrations recommended by the manufacturer. Incubation was performed for 30 min. Cells were then washed twice in T-PBS. Corresponding secondary antibodies with fluorescent conjugates were subsequently diluted in T-PBS at the concentrations suggested by the manufacture instructions. Incubate was performed for 20 min and cells were analyzed using fluorescent microscopy or flow cytometry. The intracellular antibodies used were Oct-4, Nanog and telomerase (clone Y182, hTert: Abcam).

### Karyotypic analysis

FPB cells were sent to NeoDiagnostix, Inc. (Rockville MD) for karyotypic analysis. Cells were harvested at 70–80% confluency and resuspended in 10 microliters of colcemid per ml of media. Cells were incubated at 37°C for 3–6 hrs after which cells were resuspended in 0.5 ml medium and mixed with 0.075 M KCl to a volume of 10 ml. After incubation for 10–15 min at 37°C in a waterbath cells were resuspended to in a total of 10 ml fixative (methonal:acetic acid as 3:1). Staining with DAPI for G-banding was performed by equilibrating the slides in 0.3 M sodium citrate, containing 3 M NaCl for 5 min and subsequent addition of 2 drops of Antifade with DAPI per slide prior to visualization.

### Proteomic analysis

Conditioned media from different cell lines was sent to RayBiotech, Inc (Norcross GA) for cytokine array analysis. According to the company's sample preparation instructions, the media were changed to DMEM with 0.2% fetal calf serum. Each flask was rinsed with 10 ml of this media and refilled to 7 ml. After culture for two days, the media was removed and centrifugation at 2000 rpm for 10 minutes was performed to remove cellular debris and frozen at -70°C for shipping. The cell number in culture was used to calculate the cytokine yield (pg) per million cells. DMEM with 0.2% fetal calf serum (control media) with no cells was sent for the analysis as well.

### Differentiation

#### Adipogenic differentiation

ERC were seeded at a concentration of 4 × 10[4] cells/ml in an 8 well chamber slide (Lab-Tek, Campbell, CA) with 0.5 ml media per well. When the cells reached 100% confluence they were transferred to Adipogenic Induction Media (Cambrex, East Rutherford, NJ) and cultured for 10 days with media changes every 3–4 days. Control cells were cultured in completed DMEM media. Cells are subsequently stained with AdipoRed (Cambrex) and visualized under fluorescent microscopy.

#### Osteogenic differentiation

ERC were seeded at a concentration of 1 × 10[4] cells/ml in an 8 well chamber slide (Lab-Tek) with 0.5 ml completed DMEM media per well. After the cells adhere overnight, the medium is changed to the Osteogenic Induction media (Cambrex). Cultures were cultured for 21 days with medium changes every 3–4 days. Control cells were cultured in complete DMEM. Cells were stained with Alizarin Red (ScholAr Chemistry, West Henrietta, NY) and visualized.

#### Endothelial differentiation

ERC cells were seeded at a concentration of 1.9 × 10[4] cells/ml in an 8 well chamber slide (Lab-Tek) with 0.5 ml complete DMEM per well. After the cells were cultured overnight the media was changed to the Endothelial Induction media (Cambrex). Cells were cultured for 21 days with media changes every 3–4 days. Control cells were cultured in complete DMEM. Cells are stained with anti-CD34 and anti-CD62 (Ancell) followed by fluourescently tagged secondary antibody.

#### Neurogenic differentiation

ERC cells were seeded at a concentration of 1.6 × 10 [4] cells/ml in an 8 well chamber slide (Lab-Tek) with 0.5 ml complete DMEM. After the cells adhered overnight, the media was changed to the NPMM neural induction media (Cambrex #CC-3209) and supplemented with 1% penicillin/streptomycin, 0.2 mM glutamax (Invitrogen) and hFGA-4 (Sigma F8424, 20 ng/ml). Cultures were cultured in induction or control complete DMEM media for 21 days with media changes every 3–4 days. Cells were stained with GFAP (Sigma) and Nestin (Chemicon), conjugated goat anti-mouse antibody (Bethyl Montgomery, Texas).

#### Pulmonary epithelial differentiation

ERC were seeded at a concentration of 2 × 10 [4] cells/ml on 8 well chamber slides (Lab-Tek) with 0.5 ml complete DMEM per well. When the cells reach 100% confluency the media was changed to induction medium (SAGM, Cambrex). Cultures were cultured for 10 days with media changes every 3–4 days. Control cells were cultured in complete DMEM media alone. Cells were stained with ProSP-C (Chemicon) plus conjugated Goat Anti-rabbit (Invitrogen).

#### Hepatic/pancreatic differentiation

ERC were seeded at a concentration of 2 × 10[4] cells/ml in an 8 well chamber slide (BD Biosciences #354630) with 0.5 ml CM20 per well. After the cells adhere overnight, the medium is changed to the induction medium (Cambrex) supplemented with hepatocyte growth factor (40 ng/ml), b-FGF (20 ng/ml), hFGF-4 (20 ng/ml), SCF (40 ng/ml) (all from Sigma). Cultures were maintained for 30 days with media changes every 3–4 days. Cells were stained with antibodies to Albumin (R&D #MAB1455) and insulin and developed plus secondary goat Anti-mouse (Bethyl #A90-216F) and mouse anti-rat (Serotec), respectively.

#### Cardiogenic and myogenic differentiation

8 well chamber slides were pre-coated with fibronectin (Sigma #F2006) and ERC were seeded at a concentration of 1.9 × 10[4] cells/ml. After overnight culture adherent cells were treated with complete DMEM containing 10 μM 5-Azacytidine (Sigma) for 24 hours. Subsequently the cells were cultured for 14 days in Skeletal Muscle Growth Medium (Cambrex) supplemented with 100 ng/ml b-FGF (Sigma). Cells were stained with Alpha-Actinin (Abcam) for myocyte and Skeletal Myosin (Abcam, Cambridge MA) for skeletal myocyte. For the cardiogenic differentiation, cultures are allowed to develop for 40 days with medium changes every 3–4 days and stained with Troponin I (Abcam #AB19615) plus conjugated Goat Anti-mouse (Bethyl #A90-216F). In some experiments cells were grown as hanging drop cultures as described [28] in order to visualize beating. Briefly, 30–50 μl of cells were placed on a lid of a petri-dish (Becton Dickinson Falcon #35–3002) and 5–9 ml sterile PBS to bottom of dish to maintain a humidified environment. Beating cells were detected after 5 days.

## Results

### Isolation and cloning of cells

**Figure 1:**
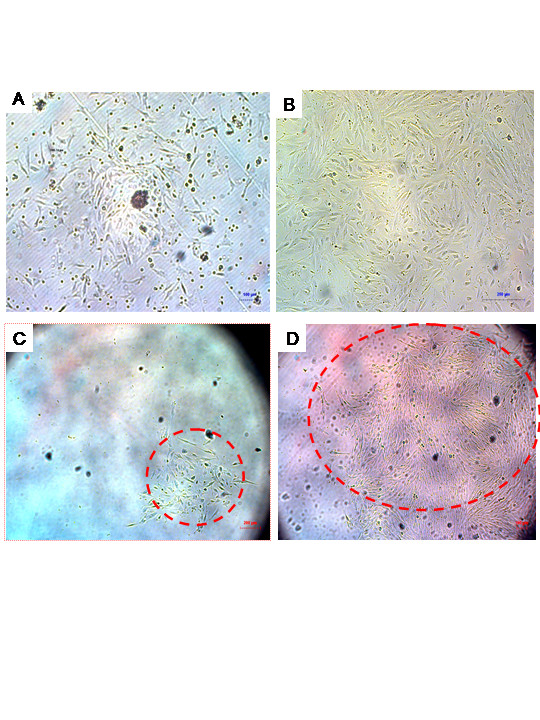
**Morphology of Cultured ERC**. A) Morphology of freshly isolated menstrual blood mononuclear cells. B) Fibroblast-like morphology of menstrual blood mononuclear cells after 2-week cell culture. C) Clonal population of menstrual cells after plating in 96 well plate 1 week after cloning. D) The same population 2 weeks after cloning.

Given the hyperproliferative state of the endometrium during preparation for implantation, and previous findings of bone marrow derived cells in the endometrium [13], we hypothesized that stem cell populations may be present in menstrual blood. To assess this possibility, 5 ml of menstrual blood was collected by urine cup-tubing method in an antibiotic containing solution. Mononuclear cells were separated by standard Ficoll methodology and grown in complete DMEM medium supplemented with 20% FCS. After overnight culture cells revealed marginal adherence to the tissue culture flask (Figure 1a). Cells were cultured for 2 weeks with media changing twice a week. At the completion of 2 week culture, an outgrowth of adherent cells with a fibroblast-like morphology was observed (Figure 1b). In order to define a clonal population of cells, we derived cell lines by single-cell plating in 96 well plates. We generated 2 cell lines, ERC-1 was derived by single-cell plating in 96 well plates, which revealed clonogenic potential (Figure 1c and 1d). The experiment was replicated with cells derived from an additional donor, which gave rise to cell line ERC-2. The cells grew at a doubling time of approximately one doubling every 19.4 hours based on quantification of cell number using microscope counting. These cells were named "endometrial derived regenerating cells" ERC based on their source and ability to differentiate into various tissues as described onwards.

### Characterization

Growth of ERC in culture appeared to be distinct from control BioE purchased cells, as well as our internally derived cord blood mesenchymal stem cells. Specifically, it appeared that ERC proliferated at a substantially faster rate as compared to the control mesenchymal cell populations. When stable cultures were compared after 20 doublings ERC had a doubling rate of 0.81 days, whereas control cells doubled in approximately 1.5–2 days (data not shown). Given the ERC appeared to have a mesenchymal-like morphology, but faster proliferative rate, we performed a series of experiments to characterize expression of mesenchymal and non-mesenchymal markers to further characterize the cells. Flow cytometric analysis revealed expression of CD9, CD44, CD29, CD41a, CD59, CD73, CD90 and CD105, while lacking CD14, CD34, CD38, CD45, CD133, STRO-1. This profile reflects a non-hematopoietic stem cell phenotype. However, some of the markers expressed are not unique to mesenchymal stem cells and are found in other tissue resident stem cells, therefore it appeared that ERC do possess some characteristics that distinguish them from mesenchymal stem cells obtained from bone marrow or cord blood (Table 1). Of notable interest, mesenchymal stem cells, including those reported in the endometrial wall itself are known to express STRO-1 [29]. ERC were negative for expression of this marker, but also negative for hematopoietic markers such as CD34, CD45 and CD133. Most interestingly the cells expressed hTERT and the embryonic stem cell marker OCT-4.

**Table 1:** Phenotypic Characterization of ERC

**Marker**	**Relevance**	**Expression**
CD14	Monocyte marker	Negative
CD34	Hematopoietic stem cell marker	Negative
CD38	Differentiating hematopoietic stem cell marker	Negative
CD45	Pan-leukocyte marker	Negative
CD133	Hematopoietic/angioblast marker	Negative
STRO-1	MSC marker	Negative
SSEA-4	Embryonic stem cell marker	Negative
Nanog	Embryonic stem cell marker	Negative
CD9	MSC marker, associated with angiogenesis^1^	Positive
CD29	Adhesion molecule on mesenchymal and hepatic stem cells^2^	Positive
CD59	Complement inhibitor protein found on MSC^3 ^and bone marrow side population CD34-stem cells^4^	Positive
CD73	Ecto-5'-nucleotidase, involved in migration of MSC	Positive
CD41a	Receptor for fibrinogen and vWF, found on MSC and platelets	Positive
CD44	Hyaluronic acid receptor found on tissue stem cells and MSC	Positive
CD90	Marker of T cells, hematopoietic and MSC	Positive
CD105	Marker of tissue and MSC	Positive
hTERT	Telomerase reverse transcriptase	Positive
Oct-4	Embryonic stem cell marker	Positive

^1 ^Kim YJ, Yu JM, Joo HJ, Kim HK, Cho HH, Bae YC, Jung JS. Role of CD9 in proliferation and proangiogenic action of human adipose-derived mesenchymal stem cells. Pflugers Arch. 2007 Aug 1.

^2 ^Chiba T, Zheng YW, Kita K, Yokosuka O, Saisho H, Onodera M, Miyoshi H, Nakano M, Zen Y, Nakanuma Y, Nakauchi H, Iwama A, Taniguchi H. Enhanced Self-Renewal Capability in Hepatic Stem/Progenitor Cells Drives Cancer Initiation. Gastroenterology. 2007 Jun 20;

^3 ^Izadpanah R, Joswig T, Tsien F, Dufour J, Kirijan JC, Bunnell BA. Characterization of multipotent mesenchymal stem cells from the bone marrow of rhesus macaques. Stem Cells Dev. 2005 Aug;14(4):440–51.

^4 ^Preffer FI, Dombkowski D, Sykes M, Scadden D, Yang YG. Lineage-negative side-population (SP) cells with restricted hematopoietic capacity circulate in normal human adult blood: immunophenotypic and functional characterization. Stem Cells. 2002;20(5):417–27.

Accordingly we sought to compare expression of proteins related to stem cell functionality between commercially available cord blood derived mesenchymal cells (BioE), cord blood mesenchymal stem cells generated by us (MYZb) and ERC. As seen in Table 2, expression of matrix metalloproteases (MMP-3 and MMP-10), cytokine growth factors (GM-CSF, PDGF-BB), and angiogenic factors (ANG-2) was constitutively present in the culture media of ERC cells in comparison to control cord blood mesenchymal stem cells which were lacking or minimally expressed these proteins. Comparable secretion of angiogenic factors such as VEGF, HGF, and EGF was observed between all cells (data not shown). Cumulatively, our characterization studies suggest that ERC share some properties of mesenchymal stem cells based on phenotype, but functionally produce factors that are unique.

**Table 2:** Proteomic Characterization of ERC Secreted Proteins

**Factor**	**BioE**	**MYZb**	**ERC-1**	**ERC-2**
MMP3	0^5^	0	**106227**	**234638**
MMP10	0	0	**5250**	**8944**
GM-CSF	0	**452**	**15630**	**972**
PDGF-BB	0	0	**12**	**61**
ANG-2	0	0	**11**	**34**

^5 ^all concentrations expressed as (pg/million cells).

### Differentiation

**Figure 2:**
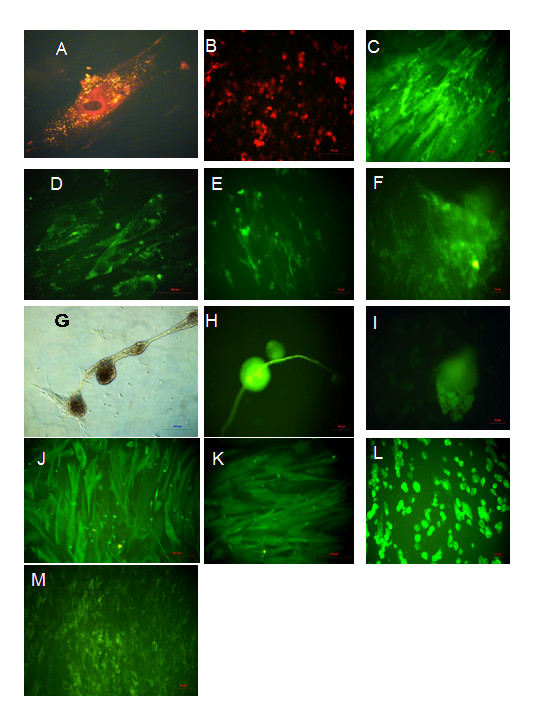
**Pluripotent Differentiation**. ERC were cultured under appropriate differentiation media as described in Materials and Methods and assessed for differentiation using the indicated staining methods. A) Adipocytic differentiation, yellow indicates lipid vacuoles stained by AdipoRed. B) Osteocytic differentiation, red indicates calcium stained by Alizarin Red. C) Myocytic differentiation, green indicates alpha actinin stain. D) Skeletal muscle differentiation, green indicates skeletal myosin stain. E) Endothelial differentiation, green indicates CD34 stain. F) Endothelial differentiation, green indicates CD62 stain. G) Hepatocytic differentiation, morphology resembles hepatic body. H) Hepatocytic differentiation, green indicates albumin stain. I) Pancreatic differentiation, green indicates insulin stain. J) Neuronal differentiation, green indicates Nestin stain. K) Neuronal differentiation, green indicates GFAP stain. L) Respiratory epithelial differentiation, green indicates Prosurfactant protein C stain. M) Cardiogenic differentiation, green indicates Troponin I stain.

Given the phenotypic, morphological, proliferation and secretory products of the ERC, we next questioned whether these cells were capable of differentiating into various lineages as described for other stem cell types. Differentiation into mesodermal (myocyte, osteocyte, endothelium, adipocyte, cardiomyocyte), ectodermal (neuronal) and endodermal (hepatic, pancreatic, respiratory epithelium) lineages was demonstrated by culturing of ERC using standard commercially available culture reagents and methodologies. As seen in Figure 2, subsequent to treatment with differentiation inducing protocols, ERC appeared to express markers of various cell lineages as detected by immunohistochemistry. ERC grown in control media in absence of differentiation stimuli did not express markers of differentiation (data not shown). In order to assess ability to form functional tissue, differentiation along the cardiomyocyte lineage was performed using the published 5-azacytidine hanging-drop method [14]. Subsequent to a 5 day culture, spontaneously contracting cells were observed. Overall, these data indicate that ERC possess the potential to differentiate into cells of all three germ lines.

### Karyotype analysis

**Figure 3:**
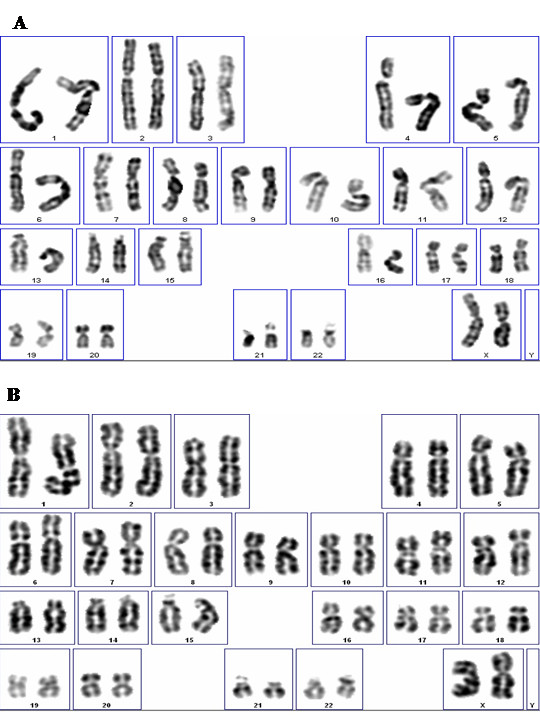
**Normality of Karyotype**. ERC were cultured for the indicated cell passage numbers and assessed for lack of karyotypic abnormalities. A. ERC at Passage 10 (38 doublings). B. ERC at Passage 18 (68 doublings). Both detailed analyses shows normal karyotypes of 46, XX.

Utility of any stem cell population depends on ability of expansion with freedom from carcinogenic potential. The rapid doubling rate of the ERC (1 doubling every 19.4 hours), as well as lack of spontaneous differentiation in absence of induction stimuli, suggests that large-scale expansion is possible for therapeutic purposes. However, in order to be useful, the cells must maintain karyotypic normality. Karyotypic analysis was performed according to routine methods, briefly, ERC were treated with colchicine and stained with DAPI for the chromosome analysis [15]. Figure 3 shows 23 pairs of chromosomes from ERC cells, after 38 and 68 cellular doublings. Twenty-one metaphases were imaged and sixteen cells were karyotyped with G-banding analysis. Detailed analysis shows a normal karyotype of 46 chromosomes with no aneuploidy, tetraploidy or other visible abnormalities. This suggests that at least at the chromosomal level ERC are capable of large scale expansion without mutagenesis.

## Discussion

Given that stem cell populations are generally associated with conditions of cellular hyperproliferation and tissue remodeling, we have examined the possibility of stem cells isolated from menstrual blood. While previously it was suggested that mesenchymal stem cells are found in endometrial tissue [13], numerous other tissues also have been found to possess endogenous mesenchymal stem cell populations which do not necessarily correlate with angiogenesis. For example cells with mesenchymal stem cell properties have been found in liver [16,17], lung [18], skin [19], pancreatic [20] and kidney tissues [21]. It was our hypothesis that the extreme angiogenesis occurring during the build-up of the endometrium would allow for specialized populations of stem cells to accumulate which could be extracted by culture of menstrual blood. We observed that the adherent fraction of menstrual blood cells could be expanded up to 68 doublings without losing karyotypic normality or developing tumorigenic potential. The cells appeared to possess some markers of mesenchymal stem cells such as CD9, CD29, CD41a, CD44, CD59, CD73, CD90, and CD105 while lacking hematopoietic stem cell markers such as CD14, CD31, CD33, CD34, CD133, and the pan-leukocyte marker CD45. Additional characteristics however make this a unique population from endometrial mesenchymal stem cells based on: 1) higher rate of proliferation compared to control cord blood derived mesenchymal stem cells; 2) lack of STRO-1 expression; 3) expression of the embryonic stem cell marker Oct-4; and 4) high expression of matrix metalloproteases. Given the ability of these cells to differentiate into tissues representative of all three germ layer components, we have named these cells "endometrial regenerative cells" (ERC).

ERC appear to have high rate of proliferation in comparison to other control mesenchymal stem cells. The positive expression of Oct-4, but negative expression of Nanog and SSEA-4 on these cells may be similar in some ways to amniotic fluid derived stem cells in that they express some but not all embryonic stem cell markers as well as telomerase reverse transcriptase [9]. One drawback of our experiments is that we did not perform functional assessment of telomerase activity using TRAP assays. These experiments are currently underway.

The possibility of ERC to be shed endometrial tissue-resident mesenchymal stem cells seems unlikely in light of several findings. Specifically, tissue stem cells of the endometrium have previously described to be bone marrow derived and to express CD34 and CD45 [22], markers which are not found on ERC. Tissue mesenchymal-like stem cells from the endometrium express STRO-1 (13a), a marker not found on ERC. Additionally, the proliferative rate of ERC (1 doubling every 19.4 hours) appears to be faster than that described for cells derived from putative uterine derived stem cells [23]. Finally, it is interesting that culture of ERC with specific "differentiation media" was able to generate cells of all three germ lines, something which has not been reported for endometrial tissue stem cells. One possible explanation for the pluripotency of ERC may be that these cells have some relationship to the "circulating oocyte progenitors" described by Tilly's group. Specifically, it was reported that bone marrow derived cells have the potential to transdifferentiate into oocyte precursors and that the presence of these cells in peripheral blood and bone marrow fluctuated with menstrual phase [24]. We are in the process of assessing ERC for expression marker's reported to be found on circulating oocyte precursors such as Vasa, Dazl and Stella. Another possibility is that ERC are involved in the angiogenesis phase of the menstrual cycle and contribute to the high level of tissue remodeling. In agreement with this hypothesis is the high level of matrix metalloprotease and growth factor production in comparison to control mesenchymal stem cell lines.

Regardless of biological significance, ERC appear to possess numerous advantages compared to other stem cell sources that make them attractive of future investigation. Firstly, the ease of collection of ERC allows for the creation of patient-specific banking. Given that the cells are expandable, as well as possessing ability to differentiate into various tissues, the cells can not only be banked until future use, but can also be expanded and pre-differentiated into various tissues so that patient-specific tissues are "on standby' and ready for use when needed. Other stem cell sources such as bone marrow and adipose tissue do not allow for such wide-spread expansion and ease of collection. Secondly, the finding that the cells can be expanded for 68 doublings without evidence of karyotypic or functional abnormalities implies that from one starting cell enough cells theoretically can be produced to treat every human being in the world. This relatively unlimited potential allows for generation of unique cell lines that can be transfected with different genes to induce specific effects. For example, cell lines can be engineered with angiogenic agents [25], neurotrophic factors [26], or to express insulin [27]. Lastly, ERC appear to have several-fold higher expression of matrix metalloproteases as compared to stem cells of other lineages. Physiologically, it is known that major remodeling of tissue is associated with the process of menstruation. Given the potential role of these cells in remodeling the endometrium, it may be reasonable to suggest that these cells are useful for treatment of fibrotic conditions such as cirrhosis in which regenerative cells with tissue degradation activities are desired. These possibilities are currently under investigation by our laboratory.

In conclusion, we have discovered a novel stem cell source from the menstrual blood that is easily accessible, highly expandable in vitro, and possesses pluripotency. This cell population may become a practical solution of choice for autologous stem cell therapy.

## Competing interests

NHR and TEI are shareholders and management of Medistem Laboratories (mdsm.ob). HW, WG, VB, and KWC are consultants for Medistem and have received payments or other considerations for their efforts. Patent applications have been filed covering composition of matter and use of ERC which are assigned to Medistem. Medistem is funding the processing charge of the current manuscript and owns all rights to know-how, intellectual property and patent applications related to this work.

## Authors' contributions

XM, JZ, AR, ZY, JJ, and NHR conceived, designed  and performed tissue culture experiments, flow cytometric evaluation,  and immunohistochemistry experiments. TEI, HW, WG, VB, KWC, BT provided   input on optimizing protocols, data interpretation, writing of the  manuscript and repeated experiments.

## References

[B1] Matikainen T, Laine J Placenta-an alternative source of stem cells. Toxicol Appl Pharmacol.

[B2] Gallo P, Condorelli G (2006). Human embryonic stem cell-derived cardiomyocytes: inducing strategies. Regen Med.

[B3] Zhang SC, Li XJ, Austin Johnson M, Pankratz MT Human embryonic stem cells for brain repair?. Philos Trans R Soc Lond B Biol Sci.

[B4] Swijnenburg RJ, Tanaka M, Vogel H, Baker J, Kofidis T, Gunawan F, Lebl DR, Caffarelli AD, de Bruin JL, Fedoseyeva EV, Robbins RC Embryonic stem cell immunogenicity increases upon differentiation after transplantation into ischemic myocardium. Circulation.

[B5] Lees JG, Lim SA, Croll T, Williams G, Lui S, Cooper-White J, McQuade LR, Mathiyalagan B, Tuch BE (2007). Transplantation of 3D scaffolds seeded with human embryonic stem cells: biological features of surrogate tissue and teratoma-forming potential. Regen Med.

[B6] Edwards RG (2004). Stem cells today: Bone marrow stem cells. Reprod Biomed Online.

[B7] Harris DT, Badowski M, Ahmad N, Gaballa MA (2007). The potential of cord blood stem cells for use in regenerative medicine. Expert Opin Biol Ther.

[B8] Parker AM, Katz AJ (2006). Adipose-derived stem cells for the regeneration of damaged tissues. Expert Opin Biol Ther.

[B9] De Coppi P, Bartsch G, Siddiqui MM, Xu T, Santos CC, Perin L, Mostoslavsky G, Serre AC, Snyder EY, Yoo JJ, Furth ME, Soker S, Atala A (2007). Isolation of amniotic stem cell lines with potential for therapy. Nat Biotechnol.

[B10] Girling JE, Rogers PA (2005). Recent advances in endometrial angiogenesis research. Angiogenesis.

[B11] Gargett CE (2007). Uterine stem cells: What is the evidence?. Human Reproduction Update.

[B12] Schmid MC, Varner JA Myeloid cell trafficking and tumor angiogenesis. Cancer Lett.

[B13] Du H, Taylor HS (2007). Contribution of bone marrow-derived stem cells to endometrium and endometriosis. Stem Cells.

[B14] http://www.biolcell.org/boc/097/0197/boc0970197.htm.

[B15] Freshney R Ian (1987). Culture of Animal Cells – A Manual of Basic Technique.

[B16] Herrera MB, Bruno S, Buttiglieri S, Tetta C, Gatti S, Deregibus MC, Bussolati B, Camussi G (2006). Isolation and characterization of a stem cell population from adult human liver. Stem Cells.

[B17] Laurson J, Selden C, Clements M, Mavri-Damelin D, Coward S, Lowdell M, Hodgson HJ (2007). Putative human liver progenitor cells in explanted liver. Cells Tissues Organs.

[B18] Majka SM, Beutz MA, Hagen M, Izzo AA, Voelkel N, Helm KM (2005). Identification of novel resident pulmonary stem cells: form and function of the lung side population. Stem Cells.

[B19] Terunuma A, Kapoor V, Yee C, Telford WG, Udey MC, Vogel JC (2007). Stem cell activity of human side population and alpha6 integrin-bright keratinocytes defined by a quantitative in vivo assay. Stem Cells.

[B20] Chase LG, Ulloa-Montoya F, Kidder BL, Verfaillie CM (2007). Islet-derived fibroblast-like cells are not derived via epithelial-mesenchymal transition from Pdx-1 or insulin-positive cells. Diabetes.

[B21] Challen GA, Bertoncello I, Deane JA, Ricardo SD, Little MH (2006). Kidney side population reveals multilineage potential and renal functional capacity but also cellular heterogeneity. J Am Soc Nephrol.

[B22] Cho NH, Park YK, Kim YT, Yang H, Kim SK (2004). Lifetime expression of stem cell markers in the uterine endometrium. Fertil Steril.

[B23] Gargett CE (2007). Uterine stem cells: What is the evidence?. Human Reproduction Update.

[B24] Johnson J, Bagley J, Skaznik-Wikiel M, Lee HJ, Adams GB, Niikura Y, Tschudy KS, Tilly JC, Cortes ML, Forkert R, Spitzer T, Iacomini J, Scadden DT, Tilly JL Oocyte generation in adult mammalian ovaries by putative germ cells in bone marrow and peripheral blood. Cell.

[B25] Mei SH, McCarter SD, Deng Y, Parker CH, Liles WC, Stewart DJ Prevention of LPS-Induced Acute Lung Injury in Mice by Mesenchymal Stem Cells Overexpressing Angiopoietin 1. PLoS Med.

[B26] Horita Y, Honmou O, Harada K, Houkin K, Hamada H, Kocsis JD Intravenous administration of glial cell line-derived neurotrophic factor gene-modified human mesenchymal stem cells protects against injury in a cerebral ischemia model in the adult rat. J Neurosci Res.

[B27] Lu Y, Wang Z, Zhu M (2006). Human bone marrow mesenchymal stem cells transfected with human insulin genes can secrete insulin stably. Ann Clin Lab Sci.

[B28] http://www.biolcell.org/boc/097/0197/boc0970197.htm.

[B29] García-Pacheco JM, Oliver C, Kimatrai M, Blanco FJ, Olivares EG (2001). Human decidual stromal cells express CD34 and STRO-1 and are related to bone marrow stromal precursors. Mol Hum Reprod.

